# Dental health of pediatric patients with X-linked hypophosphatemia (XLH) after three years of burosumab therapy

**DOI:** 10.3389/fendo.2022.947814

**Published:** 2022-08-15

**Authors:** Rafi Brener, Leonid Zeitlin, Yael Lebenthal, Avivit Brener

**Affiliations:** ^1^ The Endodontic Unit, Galilee College of Dental Sciences, Galilee Medical Center, Nahariya, Israel; ^2^ Azrieli Faculty of Medicine, Bar-Ilan University, Safed, Israel; ^3^ The Metabolic Bone Disease Unit, Dana-Dwek Children’s Hospital, Tel Aviv Sourasky Medical Center, Tel Aviv, Israel; ^4^ Sackler Faculty of Medicine, Tel Aviv University, Tel Aviv, Israel; ^5^ The Pediatric Endocrinology and Diabetes Unit, Dana-Dwek Children’s Hospital, Tel Aviv Sourasky Medical Center, Tel Aviv, Israel

**Keywords:** X-linked hypophosphatemia, rickets, FGF23, dental morphology, dental abscess

## Abstract

An inactivating *PHEX* gene mutation with the resultant accumulation of several mineralization-inhibiting proteins (e.g., FGF23) causes skeletal and dental morbidity in X-linked hypophosphatemia (XLH). This prospective case-control study explored the effect of burosumab, an anti-FGF23 antibody, on dental health of children with XLH. Ten children (age 4.3-15 years) with XLH underwent burosumab treatment per protocol. Assessment of their dental status at treatment initiation and after 1 and 3 years of treatment included clinical, laboratory and radiographic evaluation of rickets and dentition. Orthopantomographic examinations of ten healthy sex- and age-matched controls were selected for comparison. Coronal and pulp dimensions of a selected permanent mandibular molar were measured with Planmeca Romexis^®^ software. One year of treatment led to improvement of height z-score (*p*=0.019) and healing of the rickets (*p*<0.001) in the XLH patients, and those achievements were maintained after three years of treatment. Dental morphology of XLH patients, distinguished by increased pulp-coronal ratios compared to controls (*p*=0.002), remained larger after the first year of treatment (*p*<0.001) and did not attain the decrease expected with age after three years of treatment. Five patients had a history of recurrent dental abscesses, with three having undergone at least one episode during the year before burosumab initiation. One patient sustained recurrent abscesses throughout three years of treatment. The persistence of the unique dental morphology of XLH patients undergoing burosumab therapy, as evidenced by excessively larger pulp dimensions, supports the role of other *PHEX* gene-related local mineralization inhibitors, such as osteopontin, in the pathogenesis of dental morbidity.

## Introduction

X-linked hypophosphatemia (XLH; OMIM: 3307800) is an inherited disease caused by an inactivating mutation in the *phosphate-regulating endopeptidase homolog X-linked (PHEX)* gene (1). *PHEX*, which is a highly expressed gene in osteocytes, osteoblasts and odontoblasts, encodes an enzyme that degrades local small integrin-binding ligand N-linked glycoproteins (SIBLING proteins), such as osteopontin (OPN) (2). PHEX deficiency results in the accumulation of mineralization-inhibiting OPN and its fragments in the bone and dentin of animal models of XLH as well as in XLH patients (2–4). An intact PHEX also suppresses the production of the serum phosphatonin fibroblast growth factor 23 (FGF23) (5). The upregulation of FGF23 due to a *PHEX* mutation results in hyperphosphaturia, hypophosphatemia and decreased levels of calcitriol due to an inhibitory effect on 25-hydroxyvitamin D3 1-α-hydroxylase, with the resultant hypo-mineralization of bone and teeth (6).

Children with XLH often experience variable severity of impaired growth, rickets, bone deformations and spontaneous dental abscesses (7). Dental morbidity is a major health burden in XLH (8). The development of recurrent abscesses or sinus tracts associated with caries-free teeth of the primary and the permanent dentition is a frequent sequela (9). Other dental-related findings include delayed dental age due to delayed tooth eruption in both the primary and permanent dentition (10). Radiographically, the teeth have a distinctive morphology of very large pulp chambers (11, 12), and an abnormally increased pulp volume/tooth volume ratio (13). Teeth may also have a relatively thin enamel layer and dentinal defects, as well as short roots with root resorptions in primary dentition, poorly defined lamina dura and a hypoplastic alveolar ridge (9).

The main therapeutic strategy in XLH consists of alleviating the chronic hypophosphatemia. Until recently, patients had received oral phosphate and vitamin D supplementation. In 2018, burosumab (Crysvita^®^, Ultragenyx), a recombinant anti-FGF23 monoclonal antibody, was introduced as a treatment for XLH (14). Since its introduction, data on the beneficial effects of this treatment on children’s growth and on their biochemical profile have been accumulating, but data on the impact of this treatment on dental health are lacking. In January 2019, burosumab was approved by the Israeli healthcare basket committee, and it has become the treatment of choice for pediatric patients with XLH. In this study, we aimed to explore the effect of burosumab treatment on dental health, dentition and tooth morphology in pediatric patients with XLH.

## Methods

### Patient selection

This prospective observational study was conducted in a Pediatric Metabolic Bone Disease Unit in a tertiary medical center. The study protocol was approved by the Tel-Aviv Sourasky Medical Center Institutional Review Board (201910556), and the parents of the participants provided written informed consent. The data were handled in accordance with the principles of GCP.

Twenty pediatric patients with XLH are currently being treated with burosumab in the Pediatric Bone Clinic, Dana-Dwek Children’s Hospital, Tel-Aviv Sourasky Medical Center. The diagnosis of XLH was established by clinical, biochemical and radiographic criteria and confirmed by a *PHEX* gene mutation. All of these patients were referred to orthopantomography (OPT) at the initiation of burosumab. Excluded from this study were three adolescent girls who had completed linear growth prior to burosumab initiation, six children who were younger than three years of age at treatment initiation and could not cooperate with the dental radiography and one boy with the comorbidity of septo-optic dysplasia who had absent teeth secondary to a mid-face developmental disorder. The ten growing children and adolescents included in the analysis had completed three years of burosumab treatment.

Burosumab was administered by subcutaneous injection according to the recommended treatment protocol of one dose every two weeks. The dose was adjusted (between 0.8-2 mg/kg) to achieve a serum phosphorus level at the low end of the normal range for age and for healing the rickets (15). Oral phosphate supplement and calcitriol were discontinued one week before initiating the burosumab protocol (16).

### Control group

OPT examinations of ten sex- and age-matched children and adolescents at baseline and after one year were selected from the radiographic database of the Pedodontics Department, Galilee Medical Center. All of the children were healthy, without any known medical condition or use of medication which could affect bone mineralization, and they were referred to OPT as part of a routine dental examination.

### Study protocol

The study protocol consisted of multidiscipline clinic visits at different time points during burosumab therapy (at baseline and throughout one and three years of treatment). Each visit included anthropometric measurements, physical examination, laboratory evaluation and imaging (left hand, wrists, knees and OPT). The routine laboratory evaluation at each time point included serum concentrations of phosphate, calcium, alkaline phosphatase and intact parathyroid hormone.

### Auxological assessment

Growth surveillance included the anthropometric parameters of measuring weight (in light clothing and by means of a standard calibrated scale) and height (by means of a commercial Harpenden-Holtain stadiometer). Body mass index (BMI) was calculated as weight in kilograms divided by height in meters squared. Anthropometric variables (height and BMI values) were converted to sex- and age-specific z-scores according to the CDC2000 Growth Charts for the United States (17). The physical examination of the patients included pubertal stage assessment, which was performed according to Tanner and Marshall staging by a pediatric endocrinologist (18, 19).

### Rickets severity score

Rickets severity was assessed with the ten-point Thacher Rickets Severity Score (RSS) (20) for radiographs of wrists and knees for evaluating the degree of growth plate widening, metaphyseal fraying and cupping and the proportion of the growth plate affected by disease. The score rose in half-point increments from zero (normal) to 10 points (severe). Skeletal X-ray imaging was interpreted by a metabolic bone disease specialist (LZ).

### Bone age assessment

Skeletal maturation was assessed by a pediatric endocrinologist (AB) who examined the left hand and wrist radiograph according to the method of Greulich and Pyle (21). The difference between bone age and chronological age was calculated at each time point.

### Assessment of dental health and morphology

The patients’ parents were queried on the history of dental abscesses, specifically, age at first abscess and occurrence of dental abscesses during the year prior to each clinic visit. In addition, the children were referred to an OPT examination at each of the study time points. All OPT examinations of patients and controls were saved as high-resolution JPEG files and imported to Planmeca Romexis Compare^®^ software (version 3.8.1R) for analysis. OPT images were adjusted for brightness, contrast and sharpness when needed. The patients’ dental age was assessed radiographically by an endodontic specialist (RB) who used a well-accepted dental development scale (22). The difference between dental age and chronological age was calculated at each time point.

**Figure 1:**
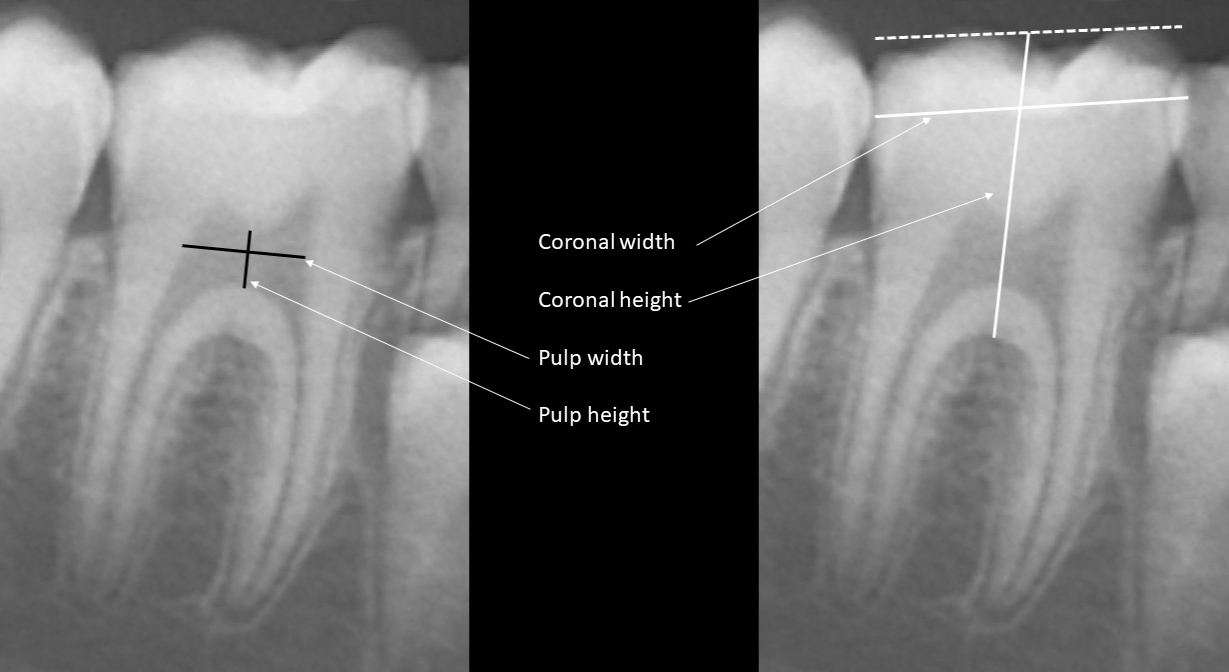
Coronal and pulp height and width were measured as follows: *coronal height* from the middle of the occlusal surface to the roof of the furcation, *coronal width* from the most distal to the most mesial points of the crown, *pulp chamber height* from the lowest point of the pulp chamber ceiling to the highest point of the pulp chamber floor and *pulp chamber width* by drawing a perpendicular line through the middle of pulp chamber height from the mesial to the distal walls of the pulp chamber.

Deciduous and permanent teeth were identified according to the Federation Dentaire Internationale Numbering System, which is a two-digit notation methodology (23). Tooth number 46, a permanent right mandibular molar, was selected for coronal and pulp chamber dimensions analysis according to this numbering system (24). Measurements were performed by the ruler tool of the Planmeca Romexis Compare^®^ software as follows (**Figure 1**): coronal height from the middle of the occlusal surface to the roof of the furcation, coronal width from the most distal to the most mesial points of the crown, pulp chamber height from the lowest point of the pulp chamber ceiling to the highest point of the pulp chamber floor and pulp chamber width by drawing a perpendicular line through the middle of pulp chamber height from the mesial to the distal walls of the pulp chamber.

The following ratios were calculated as indicators for the proportions between the pulp and the corona of the tooth: pulp-coronal height ratio (pulp height/coronal height) and pulp-coronal width ratio (pulp width/coronal width). All measurements for each patient and control were the means of two independent assessments performed by RB, and inter-operator variability was less than 3%.

### Statistics

The data were analyzed with the Statistical Package for the Social Sciences software version 27 (SPSS Inc., Chicago, IL, USA). All statistical tests were two-sided. The Kolmogorov-Smirnov test and the Shapiro-Wilk test were applied to test the normal distribution of continuous parameters. The data are expressed as means ± standard deviations (SD) for normally distributed variables and median and interquartile range (IQR) for abnormally distributed variables Pearson’s chi-square test or Fisher's exact test was performed to compare the distribution of categorical variables. The paired t-test was used for comparing the means of variables at different time points. Pearson or Spearman correlations were applied to determine correlations between variables. A *p* value of ≤ 0.05 was considered significant.

## Results

At the initiation of the burosumab protocol, the ten study patients (four boys and six girls) with XLH were 8.8 ± 3.8 years old (range 4.3-15.0). Six of them were prepubertal and four were in mid-puberty (Tanner stages 2-4). They were diagnosed with XLH at a mean age of 1.9 ± 1.3 years (range 0.5-5 years) and had been treated with conventional therapy for 7.0 ± 4.2 years. Six (60%) of the ten patients had a family history of XLH, and they had been diagnosed at a younger age than those without affected relatives (1.3 ± 0.8 vs. 2.8 ± 1.5 years, *p* < 0.001, respectively).

The baseline clinical, laboratory and radiographic characteristics of the patients at burosumab initiation are presented in **Table 1**. The mean ± SD height z-score was -1.61 ± 0.75, with five (50%) patients below the 3^rd^ percentile for height. All of the patients had phosphate levels below the lower limit of the normal range for age in combination with increased alkaline phosphatase levels. The RSS ranged from 1 to 3. Baseline bone and dental ages were significantly correlated with chronological age (r = 0.990, *p* < 0.001 and r = 0.985, *p* < 0.001, respectively).

**Table 1:** Clinical, laboratory and radiographic baseline characteristics of ten patients with X-linked hypophosphatemia (XLH) at initiation of burosumab treatment.

Case	Sex	Age (years)	*PHEX* gene mutation	Height (z-score)	BMI (z-score)	Serum phosphate (mg/dL)	Alkaline phosphatase (U/L)	PTH (pg/mL)	RSS	Bone age (years)	Dental age (years)
1	F	4.2	c.1328G>A(het); p.Arg443His	-0.88	0.62	3.4	499	13.8	2	2.5	4.5
2	F	4.3	c.1783A>T(het); pLys595*	-2.63	2.13	2.5	636	65.6	3	3.50	4.5
3	M	5.6	c.146delIT; p.His487Glnfs*28	-2.01	1.58	4.7	471	38.8	3	5.5	6.5
4	M	6.3	c.565C>T(het); pGin189X(STOP), mosaicism50%	-0.19	0.84	3.4	351	43.2	1	6	6.5
5	F	8.0	c.146delIT; p.His487Glnfs*28	-1.48	0.39	4.0	335	38.8	2	8.8	7.5
6	F	9.5	Not found	-0.85	0.49	2.2	521	74.2	3	9	11.5
7	M	9.9	c.488C>A(hemi); p.Ser163*(STOP)	-1.40	1.34	2.6	667	21.3	3	10	11.5
8	F	11.8	c.1645C>T; p.R549*	-2.42	1.77	2.6	543	32.0	3	11	12.5
9	F	13.3	c.1735G>A; p.Gly579Arg; rs875989883	-2.07	-0.91	2.8	609	46.6	2.5	13.5	13.5
10	M	15.0	c.1735G>A; p.Gly579Arg; rs875989883	-2.18	0.16	2.0	241	14.9	3	16	16.5

F, Femal; M, Male; BMI, Body mass index; PTH, parathyroid hormone; RSS, rickets severity score.

The clinical and radiographic characteristics of XLH patients during three years of burosumab treatment are presented in **Table 2**. Their mean height z-score increased significantly during the first year of treatment (*p* = 0.019), with stabilization after three years of treatment (*p* = 0.722). The mean BMI z-score did not change significantly throughout the three years of treatment. All patients demonstrated healing of the rickets with normalization of RSS during the first year of treatment, and the three-year radiographic follow-up demonstrated preservation of these achievements. Radiographic healing of rickets was consistent with the decrease in alkaline phosphatase levels after one and three years of treatment (*p* = 0.004 and *p* = 0.386 respectively).

**Table 2:** Three-year surveillance of ten burosumab-treated X-linked hypophosphatemia (XLH) patients.

	Baseline	1 year	3 years	*P^1^*	*P^2^*
**Age, years**	8.8 ± 3.8	9.8 ± 3.8	11.8 ± 3.8	** **	** **
**Burosumab dosage, mg/kg/month**	2.09 ± 0.96	2.28 ± 1.20	2.03 ± 1.23	**0.033**	0.952
**Laboratory evaluation**					
**Serum phosphate, mg/dL**	3.03 ± 0.85	3.58 ± 0.50	3.63 ± 0.53	0.125	0.967
**Serum calcium, mg/dL**	9.57 ± 0.38	9.59 ± 0.39	9.62 ± 0.26	0.831	0.356
**Serum alkaline phosphatase,**	487.3 ± 140.0	337.4 ± 142.6	300.4 ± 127.8	**0.004**	0.386
**U/L**					
**Serum PTH, pg/mL**	38.92 ± 20.00	36.33 ± 21.28	34.20 ± 14.07	0.731	0.374
**Anthropometric measurements**					
**Height, z-score**	-1.61 ± 0.79	-1.45 ± 0.81	-1.42 ± 0.88	**0.019**	0.722
**Weight, z-score**	-0.29 ± 0.70	-0.12 ± 0.69	0.17 ± 0.75	0.466	0.092
**BMI, z-score**	0.84 ± 0.82	0.82 ± 0.85	1.07 ± 0.73	0.926	0.111
**Dental health**				
**Patients with dental abscesses**	3 (30)	1 (10)	1 (10)	0.582	1
**Radiological evaluation**				
**Rickets severity score, median**	3 [1-3]	0 [0-1]	0 [0]	**<0.001**	0.952
**Δ bone age, years**	-0.27 ± 0.70	-0.13 ± 0.51	-0.07 ± 0.46	0.419	0.087
**Δ dental age, years**	0.65 ± 0.74	0.47 ± 1.07	0.95 ± 1.35	0.243	0.115
**Tooth morphology**					
**Pulp-coronal height ratio**	0.32 ± 0.07	0.33 ± 0.08	0.29 ± 0.05	0.287	**0.009**
**Pulp-coronal width ratio**	0.48 ± 0.11	0.45 ± 0.11	0.40 ± 0.11	0.482	0.084

BMI Body mass index, PTH parathyroid hormone.

Data are expressed as mean ± standard deviation (SD), median [range] or number (percent).

Δ bone age represents the difference between bone age and chronological age.

Δ dental age represents the difference between dental age and chronological age.

P^1^ represents the paired t-test comparison between the baseline value and after one year of treatment.

P^2^ represents the paired t-test comparison between the values after one and three years of treatment.

Bold indicates significant.

**Figure 2:**
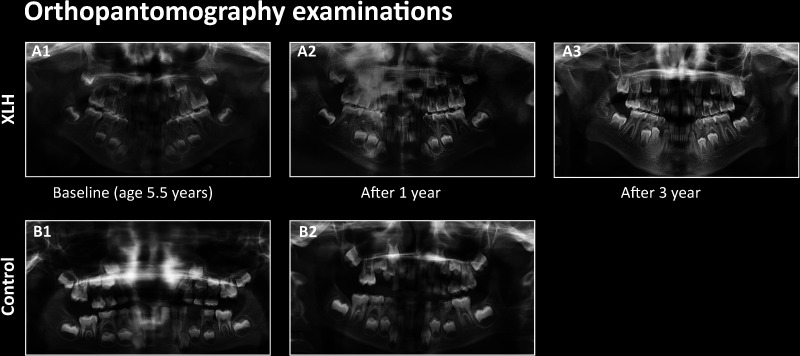
Orthopantomography (OPT) of patient #3 at burosumab initiation **(A1)**, after one year **(A2)** and after three years of treatment **(A3)**, compared to the OPT of a healthy age-matched boy at baseline **(B1)** and after one year **(B2)**.

**Figure 3:**
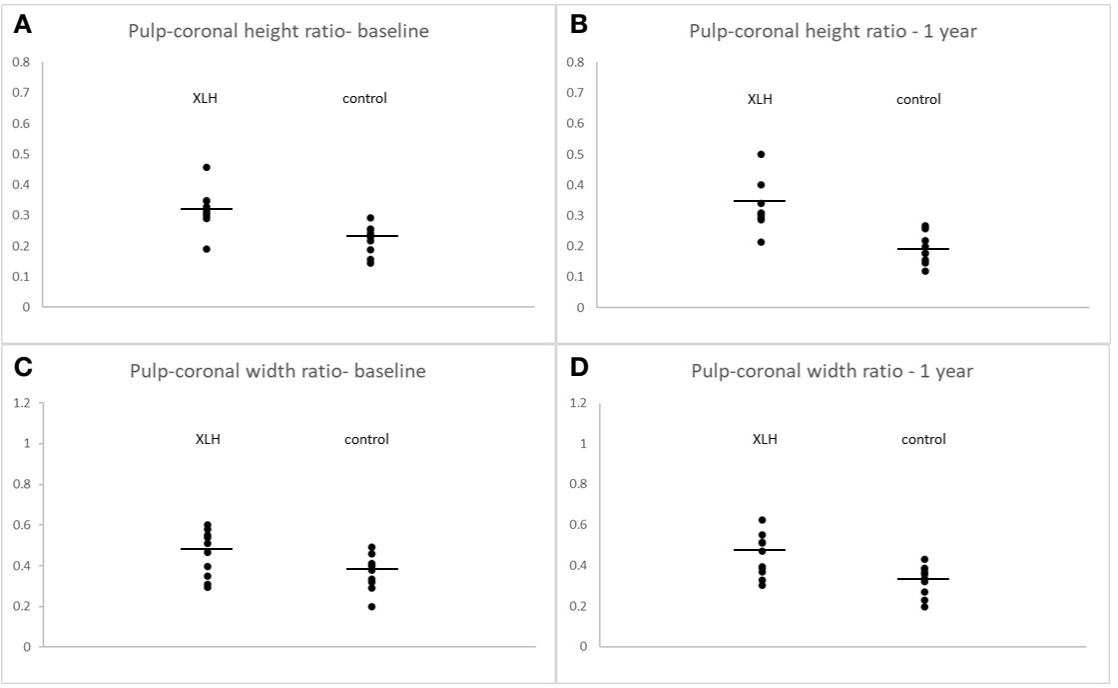
Individual values of pulp-coronal height ratios at burosumab initiation **(A)** and after one year **(B)**. Pulp-coronal width ratios at burosumab initiation **(C)** and after one year **(D)** in X-linked hypophosphatemia patients and their controls. Lines indicate mean values. All comparisons were statistically significant at a p value of ≤ 0.05.

Five of the ten study patients (50%) had a history of recurrent dental abscesses in primary teeth. The mean age at first abscess was 4.3 ± 1.0 years, and three of them had at least one episode during the year prior to burosumab initiation. One patient sustained recurrent abscesses involving a deciduous molar tooth (patient #3 **Table 1**), and no desirable serum phosphate levels were achieved by the administration of the recommended burosumab dose, necessitating a gradual dose increase up to 50 mg every 10 days (4.8 mg/kg/month). Since remission in that child’s abscesses had not been obtained after reaching the target phosphate levels, the affected deciduous tooth was extracted, revealing a resorbed root. The OPT evaluations of that patient at treatment initiation and those after one year of treatment are presented in (**Figures 2A1, A2**), respectively, compared to the OPT of his sex- and age-matched control (**Figures 2B1, B2**). (**Figure 2A, 3**) presents this patient’s OPT after three years of burosumab treatment.

(**Figure 3**) illustrates the comparative tooth compartment ratios between XLH patients and controls at baseline and after one year. At baseline, the pulp-coronal height ratio of the XLH patients was significantly greater than that of the controls (0.32 ± 0.07 vs. 0.22 ± 0.05, *p* = 0.002) and remained unchanged after one year (0.33 ± 0.08 vs. 0.19 ± 0.05, *p* < 0.001). The pulp-coronal width ratio of the XLH patients was also greater than that of the controls at baseline (0.48 ± 0.10 vs. 0.38 ± 0.11, *p* = 0.048), and this difference persisted after one year (0.45 ± 0.11 vs. 0.33 ± 0.07, *p* = 0.010). After three years of treatment with burosumab, the pulp-coronal height ratio of the XLH patients decreased (0.29 ± 0.05, *p* = 0.009), while their pulp-coronal width ratio remained unchanged (0.40 ± 0.11, *p* = 0.084). Also after three years of treatment, when their mean age was 11.9 ± 3.9 years, the XLH patients still had larger pulp-coronal height and width ratios than those of the control group whose mean age was 9.6 ± 3.3 years (*p* < 0.001 for both parameters).

## Discussion

This prospective study is the first to report that the unique dental morphology of excessively larger pulp dimensions in patients with XLH persisted during three years of burosumab treatment. In line with previous reports, we also demonstrated that burosumab treatment normalized phosphate levels, contributing to the healing of rickets and to the improvement of linear growth of pediatric patients with XLH during the first year of treatment, with maintenance thereafter (25, 26). Pediatric patients with XLH reportedly exhibited a delayed eruption of both deciduous and permanent teeth (27). Contrarily, none of our ten patients had demonstrated any remarkable delay in dental age prior to burosumab initiation, as assessed by the timing of permanent teeth eruption. Moreover, the difference between dental age and chronological age did not change significantly throughout the years of burosumab treatment.

At baseline, both the dental age and the bone age of our patients demonstrated a significant correlation with chronological age, with the bone age being relatively delayed. This gap gradually diminished during the three years of burosumab treatment, indicating that linear growth improvement was accompanied by a catch-up in bone age. Mao et al. (28) recently observed that a better understanding of the extent and duration of catch-up growth during the first years of burosumab treatment, which mainly stems from the healing of rickets, may assist in predicting adult height of children embarking upon this treatment.

Dental morbidity is a major health burden in XLH patients who may sustain recurrent spontaneous abscesses in deciduous and permanent teeth (4, 29), increased vulnerability to endodontic infections and recurrent periodontitis (30). Dental morbidity during childhood can adversely affect the child’s behavior, nutrition and quality of life (31). The need for frequent dental care services can become major mental and economic burdens, and may be out of reach for pediatric patients whose families have lower socioeconomic positions (32). Notably, poor oral health that leads to the development of chronic periodontitis has been associated with the development of cardiovascular complications (33). These considerations mandate preventive strategies aimed at amending the dental phenotype of XLH patients already during early life.

Clinical studies report on a periodontal disease in chronic hypophosphatemia, related to aplasia or hypoplasia of the cementum. These could prevent normal attachment of ligament fibers and favor faster progression of periodontal disease with early loss of teeth (34). Evidence is lacking on the impact of burosumab treatment on periodontal condition and further studies with clinical and radiographic evaluation are warranted.

The morbidity associated with XLH is caused by the under-mineralization of highly mineralized tissues, such as bone and teeth, leading to the clinical spectrum of rickets and osteomalacia of bone and odontomalacia of teeth (29). Treatment consisting of either the conventional therapy of phosphate supplement combined with vitamin D analogs or the novel anti-FGF23 antibody, burosumab, is aimed at increasing the level of circulating serum phosphate (14, 35), not necessarily at affecting local phosphate levels (36).

OPN is a major noncollagenous protein of the extracellular matrix that regulates the mineralization process (4). Studies which examined bone and dental tissues of patients with XLH revealed differences in the distribution of OPN accumulation, thus leading to divergent defect in mineralization. In XLH bone, abundant OPN and OPN fragments reside in the pericellular hypomineralized halo area around osteocytes while in XLH tooth, most OPN was associated with the demineralized protein extracts in the dentin extracellular matrix. This dissimilarity between bone and tooth may reflect variances in mineral metabolism between these two tissues, where the mineralized matrix of dentin is more static and does not remodel as it does in bone (4).

Conventional therapy was found to be inadequate for improving biochemical and radiological features of the disease (37), while burosumab was reported as being successful in normalizing and stabilizing phosphate levels in most of the patients (16). Treatment aimed at increasing the serum phosphate levels and thereby improving dentin mineralization and decreasing the rate of endodontic infections was reportedly a beneficial therapeutic strategy (38). However, the demonstration of OPN accumulation in XLH highly mineralized tissue may bring into question the ability of burosumab treatment to fully rescue the defect.

The phosphate levels of most XLH patients eventually normalize while they undergo the recommended dose of burosumab. However, there are patients with poorer response to the medication in whom a higher dose is required for stemming the ongoing loss of phosphate in the urine. Laurent et al. (39) recently suggested that possible explanatory mechanisms for drug resistance may involve anti-drug antibodies, but laboratory measurements of those antibodies are not yet available and the authors did not recommend their assessment as part of routine clinical practice. Our patient #3 suffered from persistent dental abscesses in deciduous molars concomitant with phosphate levels below the desirable range under the maximal recommended dose of burosumab. Administration of burosumab injections every 10 days instead of every 14 days achieved improvement in his serum phosphate levels. However, dental abscesses recurred until the tooth was extracted, suggesting that the abnormal enamel and primary dentin structure that had already been formed could not be counteracted by an increase in serum phosphate intake.

The normal development of a tooth comprises a gradual formation of the secondary dentin, thus reducing the pulp chamber dimension. The pulp-to-coronal ratio is therefore expected to decrease with age (40). The teeth of XLH patients are distinguished by abnormally enlarged pulp chambers, morphologically interpreted as a younger stage of dental development. We found that this pathological phenotype persisted after the patient had received one year of burosumab treatment, as demonstrated by larger pulp-to-coronal height and width ratios of XLH patients compared to healthy controls. Unfortunately, OPT examinations of healthy pediatric controls were not available for comparison at the three-year time point and references in the literature are available only for adults. Still, the pulp-coronal ratios of patients did not change significantly.

The frequency of dental abscesses in our ten study patients decreased significantly throughout the three years of burosumab treatment, with only one exception. Various physiological and behavioral alterations may contribute to this favorable result, including an increased awareness to dental hygiene on the part of the caregivers. Dental abscesses and persistent periodontal disease have been commonly reported in children during the first 64 weeks of burosumab treatment (14). A study in Hyp mice reported that the administration of anti-FGF23 antibodies did not improve mineral density of the dentin (41). This may support the conclusion that the decrease observed in dental abscess frequency in our patients does not stem from an improved local mineralization.

The ongoing accumulation of the defective PHEX non-degraded proteins in the extracellular matrix may affect the tissue structure, with the resultant unresolved morbidity. In the XLH dentin, OPN accumulates in the calcified regions and along tubules (42). Studies that evaluated the histomorphometry of XLH teeth reported abnormal dentin structure characterized by cracks and fissures extending to the enamel that enabled bacterial invasion into the pulp and led to necrosis and periapical complications (43). A disturbed mineralization pattern was also identified around odontoblast processes, which are regions of dentin where OPN has been shown to accumulate (42). This contributor to dental pathogenesis is not directly affected by burosumab therapy, and that characteristic may provide the explanation for its persistence in pathologic dental morphology.

The present study has several limitations and strengths that bear mention. A major limitation is the small study population, a factor mainly due to the rare occurrence of the disease and the strict inclusion of patients who underwent OPT at all three time points. The major strengths of our study are its prospective design, the multidisciplinary collaboration in the treatment and surveillance of our patients, and the uniformity of growth measurements, physical examinations and interpretations of the radiographic imaging studies by trained medical personnel throughout the study.

In conclusion, burosumab treatment normalized phosphate levels, healed rickets and improved linear growth. Regrettably, the dental morphology of XLH patients as evidenced by excessively larger pulp dimensions did not exhibit the desired decrease in the pulp dimensions expected with age. Our findings suggest that PHEX-related local mineralization inhibitors, such as osteopontin, play a critical role in dental morbidity. Future therapeutic modalities should target the local hypomineralization defects.

## Data availability statement

The raw data supporting the conclusions of this article will be made available by the authors, without undue reservation.

## Ethics statement

The studies involving human participants were reviewed and approved by Tel Aviv Sourasky Medical Center. Written informed consent to participate in this study was provided by the participants’ legal guardian/next of kin.

## Author contributions

Authors RB prepared the first draft of the paper. Author AB designed the study, was responsible for statistical analysis of the data, prepared the first draft and is guarantor. Authors RB, LZ, YL, and AB contributed to the experimental work. All authors revised the paper critically for intellectual content and approved the final version. All authors agree to be accountable for the work and to ensure that any questions relating to the accuracy and integrity of the paper are investigated and properly resolved.

## Acknowledgments

The authors thank all of the participating patients and their families and the multidisciplinary team that cares for XLH patients treated in the Metabolic Bone Clinic. We wish to thank Esther Eshkol for editorial assistance. Parts of these data were presented in The International Conference on Children’s Bone Health (ICCBH), July 2022, Dublin, Ireland.

## Conflict of interest

The authors declare that the research was conducted in the absence of any commercial or financial relationships that could be construed as a potential conflict of interest.

## Publisher’s note

All claims expressed in this article are solely those of the authors and do not necessarily represent those of their affiliated organizations, or those of the publisher, the editors and the reviewers. Any product that may be evaluated in this article, or claim that may be made by its manufacturer, is not guaranteed or endorsed by the publisher.
